# The Discipline of Medical Schools

**Published:** 1888-09-15

**Authors:** 


### The Discipline of Medical Schools.

Medical students have for a long time enjoyed a very unenviable reputation. They are supposed to be idle, ill-mannered, and, in general, "rowdy" in their behaviour, even when they are not charged with absolute cruelty to the patients entrusted to them during their hospital studies. This is the popular estimate of them. The popular estimate of the medical practitioner is, on the contrary, that he is a thoughtful, courteous, and competent gentleman, to whom the issues of life and death may fitly be confided. How such a transformation can be effected by the mere receipt of a qualification or licence to practice, no one, however ready to believe that increased responsibility brings increased fitness to bear it, can satisfactorily explain. As a matter of fact, no such explanation is needed. The majority of medical students are not idle, nor rough, nor brutal, and their development into the doctors we know is a very natural process. But in the student days it is the black sheep who come most before the public, the men who, if they ever gain a degree at all, make such poor use of it that the public in general knows nothing of them. They are of no account, and their deeds and misdeeds, unless they so utterly forget the honour of their profession as to sink into criminal practice, do not affect the general good opinion in which doctors are justly held, because they are so utterly unknown to any large section of the public.

But these black sheep are somebody's sons; and their parents and guardians, naturally unwilling to blame their own wards or children, cry out that the medical schools are not properly conducted, that the authorities of these are anxious only to show a large matriculation roll and receive a large sum in fees; while, those objects being secured, they take no further interest in their students. These dissatisfied fathers ask for stricter supervision, they demand that the lecturers at medical colleges shall become preparatory schoolmasters, and the other authorities nursemaids; they think that the doctor in his chrysalis state should be wrapped up in a moral cocoon, from which he shall emerge a miracle of wisdom and virtue, ready, without previous experience of independent and responsible life, without the strength that freedom and knowledge give, to hold his own in the world to its profit and his own prosperity. Hearing, as everyone who comes in contact with medical students and their friends must occasionally do, such protests as these, we ask first, if the complaint is just, and, secondly, if the proposed remedy is wise or practicable?

Having been at the trouble to invite the opinions held by the deans, and other responsible authorities of the principal medical schools in the kingdom on the subject, we can speak with the authority given by the full and courteous answers with which most of these gentlemen have favoured us.

In the first place, do the authorities of the medical schools exercise any supervision over the students attending them? The unanimous answer is that they do. To many of the colleges and hospitals there are residential halls attached, in which such students as live there are kept under a supervision as rigorous as would be exercised over young men of their years at home; and it must be known to many of our readers how often youths who, though well intentioned, know themselves to be weak in face of temptation, go to live in these institutions for the very purpose of being helped by the wholesome restraint of their rules, and the influence of the steadier men who most gladly congregate in them. But it would be obviously unreasonable, and in fact, impossible to insist on all students living in these halls. Many of them have homes in the town where they are studying, and considerations of economy forbid their residing elsewhere. The same considerations affect many of the most industrious and intelligent of the student class, who can afford to pay for their professional studies only by exercising the severest thrift; to them the moderate and reasonable charges of the Hall are wholly prohibitive. Some students—only a few, indeed, but still a minority that must be considered—are married, and would certainly object to being separated from their wives and children, even though the infant tongues occasionally announce, "Mamma, daddy's plucked again."

It must be remembered that our medical students are not all boys, though it is to be regretted that so many parents send their sons direct from school to college, whereas a year spent in those preliminary scientific studies, which do so much to mould the temper of the mind, apart from their direct benefit to the medical student and practitioner, might either form a valuable preparation for their studies or prove their unfitness for the profession they think of entering—thus saving much time, money, and disappointment. Still, we have seen a grey-haired gentleman, who already had the right to put the letters "D.D." after his name, stand up to receive the degree of M.B. Surely it would have been a little hard to have insisted on his submitting to the regulations, however wise and reasonable for younger, untried men, of a residential college. Allowing that this is an extreme case, it must still be insisted on that a large proportion of our medical students have more than attained their legal majority and are competent to guide their own steps; and also that the laws of any institution must be adapted to the requirements of the majority of its adherents, not to those of the minority.

While, however, it is necessary that any restraint exercised over the medical student out of class-time must be left to his option, or that of his guardians, the college authorities by no means neglect such supervision of his studies as is necessary and desirable. We publish in this number a letter from a gentleman who recommends, or rather demands, that the students shall at a certain hour assemble in a class-room and be "heard their lessons" by a master, and also that they shall not be allowed to leave the hospital except at specified hours. In the first place, this gentleman errs by confounding the medical school with the hospital. They are not identical: except in London there is no inevitable connection between any single school or a particular hospital. The student is required to produce a certificate of having gone through a certain amount of clinical study before he goes up for his final examination, but he does not, unless he is appointed to some post in connection with it, pass his student life within the hospital walls. Perhaps when rushing hastily from college to hospital or back again he wishes he did.

This certificate, however, proves that he has availed himself of the opportunities open to him for clinical study to the extent of going through the wards with his teacher a certain number of times during the session. If it does not prove that he has used them as profitably as he might have done, it fails, only to show that taking a horse to the water is equivalent to making him drink—a thing generally looked upon as beyond human power. The coming examination will, in all probability, put that point to the test, and show how much medical knowledge he has managed to imbibe; for in spite of popular notions, it is no easy thing to "cram" the average mind with those practical details of treatment about which the student is questioned in a clinical examination.

In the theoretical classes similar precautions are taken to ensure regular attendance. We may quote as an example of college regulations in this matter the first and second rules laid down for observance by lecturers at the Royal College of Surgeons at Edinburgh:—

"1. Every lecturer at this school of medicine shall ascertain, at least twenty-five times in a six months' course, and twelve times in a three months' course, the actual attendance given by his pupils.

"2. He shall do this by calling the name of each pupil at least once a-week, and enter in a regular roll-book the presence or absence of each individual; the students, of course, being kept in ignorance of the particular days on which this is to be done, excepting on the occasion of the first roll-call, which shall not be later than the fifteenth day of the session."

It is true that the ingeniously lazy student can evade the supervision the authorities of his school thus try to exercise. There is always a kind and unscrupulous friend at hand to write his name in the attendance-book, or answer "Present" when his name is called and he is not in the class-room. If the attendance is marked by the student giving his visiting card on certain days when he enters the class-room, a bribe given to the janitor will induce him to add that of an absentee to the collection. But such things as these are inevitable. Make regulations of the utmost stringency, and there will still be found men who will succeed in evading them. There are students who could not fail to become shining lights in their profession if they showed half as much energy in persuing as they display in evading their work. The impossibility of compelling men to industry against their will was felt so strongly by one professor at the Edinburgh University that he told his class at the beginning of the session the days on which he meant to call for their cards, and left it to their honour to be faithful in their attendance on the days when it was not noted. It is true that his talents and popularity were such as to render him always sure of a class-room full to overcrowding, and it might not be advisable for all lecturers to follow his example. But his action showed that he felt it impossible to convert idleness into industry by any other than moral force; and the experience of most of the deans and registrars of our medical schools will justify his action.

With regard to the "hearing of lessons" that is demanded by some parents, it should be mentioned that written class-examinations are frequently held, and those who distinguish themselves in these receive certificates, which, though they do not influence the result of the professional examinations, are eagerly sought after; while, still more frequently, the lecturer will orally question his class on the work they have recently gone through. There is no neglect of putting the student's knowledge to the test.

Many parents complain also that their sons announce so frequently that they must go up for some examination, an indispensable preliminary to which is a large fee, that they cannot but doubt the truth of the statement. Often their doubt is justifiable, but in this again the authorities of the schools are not to blame. Every school publishes a calendar or prospectus, in which the date of the various examinations and the fees for them are clearly set forth, and it is surely not too much to expect that those who are most directly interested in our student's welfare will ascertain for themselves the conditions and expense of his professional studies. If, however, they have not taken this precaution, a letter to the dean of the medical faculty, in whatever college their son may be, is almost certain to bring forth a clear and courteous reply. And, indeed, while these gentlemen hesitate long before disgracing a student in the eyes of his family by any complaint of misconduct, they are always grateful when parents will co-operate with them in establishing some surveillance over him, while there are but few of the lecturers in our colleges and universities who do not try in some fashion or other to win the personal acquaintance of those in their classes.

A certain amount of supervision, therefore, is exercised over the medical student. Is a stricter watch desirable? Surely not. He is in many cases, as we have pointed out, one who has reached man's estate, and in all he is preparing for the work, the independence, and the responsibilities of manhood. If this be so, his education must consist not merely in the storing of his brain with certain facts concerning the human body in health and disease, but in that moral and intellectual development which will enable him to do his share—a most onerous and responsible one—of the world's work with wisdom, firmness, and self-respect. These are qualities not born of servitude nor largely bred in tutelage. Freedom is the primary condition of their existence, and only by a gradually increasing amount of personal and intellectual liberty can the student be made fit for those privileges and grave responsibilities with which the possession of his diploma endows him. It is true that by some this liberty will be abused, but these form a very small minority of the whole; and if we may be allowed to conclude with a surgical metaphor, we will express our meaning by saying that because children who suffer from weakness of the spine may with advantage wear iron supports, it is not desirable to add such troublesome impedimenta to the general clothing of the human race.

